# Case Report: Inactivating PTH/PTHrP Signaling Disorder Type 1 Presenting With PTH Resistance

**DOI:** 10.3389/fendo.2022.928284

**Published:** 2022-06-30

**Authors:** Tanguy Demaret, René Wintjens, Gwenaelle Sana, Joachim Docquir, Frederic Bertin, Christophe Ide, Olivier Monestier, Deniz Karadurmus, Valerie Benoit, Isabelle Maystadt

**Affiliations:** ^1^ Centre de Génétique Humaine, Institut de Pathologie et Génétique (IPG), Gosselies, Belgium; ^2^ Unité Microbiologie, Chimie Bioorganique et Macromoléculaire (CP206/04), Institut de Pharmacie, Université Libre de Bruxelles (ULB), Brussels, Belgium; ^3^ Service de Pédiatrie, Grand Hôpital de Charleroi (GHdC), Charleroi, Belgium; ^4^ Service de Radiologie, Grand Hôpital de Charleroi (GHdC), Charleroi, Belgium; ^5^ Département de Médecine, Unité de Recherche en Physiologie Moléculaire (URPhyM), Université de Namur (UNamur), Namur, Belgium

**Keywords:** PTH1R, iPPSD1, epilepsy, parathyroid hormone, alfacalcidol, GNAS, Albright hereditary osteodystrophy, pseudohypoparathyreoidism

## Abstract

PTH resistance is characterized by elevated parathyroid hormone (PTH) levels, hypocalcemia, hyperphosphatemia and it is classically associated with *GNAS* locus genetic or epigenetic defects. Inactivating PTH/PTHrP signaling disorders (iPPSD) define overlapping phenotypes based on their molecular etiology. iPPSD1 is associated with *PTH1R* variants and variable phenotypes including ossification anomalies and primary failure of tooth eruption but no endocrine disorder. Here we report on a 10-month-old child born from consanguineous parents, who presented with mild neurodevelopmental delay, seizures, enlarged fontanelles, round face, and bilateral clinodactyly. Hand x-rays showed diffuse delayed bone age, osteopenia, short metacarpal bones and cone-shaped distal phalanges. A diagnosis of PTH resistance was made on the basis of severe hypocalcemia, hyperphosphatemia, elevated PTH and normal vitamin D levels on blood sample. The patient was treated with calcium carbonate and alfacalcidol leading to rapid bio-clinical improvement. Follow-up revealed multiple agenesis of primary teeth and delayed teeth eruption, as well as Arnold-Chiari type 1 malformation requiring a ventriculoperitoneal shunt placement. *GNAS* gene analysis showed no pathogenic variation, but a likely pathogenic homozygous substitution c.723C>G p.(Asp241Glu) in *PTH1R* gene was found by trio-based whole exome sequencing. We studied the deleterious impact of the variant on the protein conformation with bioinformatics tools. In conclusion, our study reports for the first time PTH resistance in a child with a biallelic *PTH1R* mutation, extending thereby the clinical spectrum of iPPSD1 phenotypes.

## Introduction

Parathyroid hormone (PTH) resistance is characterized by elevated plasma PTH levels, hypocalcemia, and hyperphosphatemia (1). As a consequence of hypocalcemia, patients may present with peripheral neuromuscular hyperexcitability (paresthesia of the extremities, perioral numbness, muscle cramping) and seizures (2).

Association of PTH resistance with specific features was formerly classified under the pseudohypoparathyroidism (PHP) term. PHP type 1 (PHP1) and type 2 (PHP2) are typically distinguished by the blunted cyclic adenosine monophosphate (cAMP) response to G protein activation (i.e. by exogenous PTH administration) seen in PHP1, as opposed to the normal response seen in PHP2 (1). PHP1 is further differentiated according to the presence (PHP1A, MIM #103580, and PHP1C, MIM #612462) or absence (PHP1B, MIM #603233) of Albright hereditary osteodystrophy (AHO) (3). AHO is a clinical entity encompassing heterogeneous clinical findings such as brachydactyly, rounded face, short stature, subcutaneous ossification and variable degrees of intellectual deficiency (4, 5).

PTH resistance is reported among multiple overlapping phenotypes associated with various molecular defects. In 2016, a more effective classification based on the molecular defect underlying PTH resistance was published and termed “inactivating PTH/PTHrP signaling disorders” (iPPSD, **Supplementary Table 1**) (6). iPPSD clinical diagnosis is made in the presence of major and/or minor criteria (**Supplementary Table 2**). iPPSD1 encompasses four phenotypes associated with *PTH1R* pathogenic variants (**Table 1**). Recently, PHP1B was associated with a homozygous variant in *PTH1R* in an adult patient presenting with partial seizures and intracranial calcification (7).

**Table 1:** iPPSD1: phenotypes associated with *PTH1R* pathogenic variants.

	MIM number	Inheritance	Suspected mechanism	Antenatal manifestations	Growth	Facial dysmorphism	Teeth	Skeletal features	Lab findings	Evolution
Blomstrand chondrodysplasia	215045	AR	Loss of function	Hydrops fetalis, polyhydramnios	n.a.	Midface hypoplasia, mandibular hypoplasia	n.a.	Advanced skeletal maturation, short limbs	n.a.	Death at birth or shortly thereafter
Eiken syndrome	600002	AR	Loss of function	n.a.	Short stature	n.a.	Primary failure of eruption	Delayed ossification, brachydactyly, partial sacrum agenesis	Normal calcemia, elevated or normal PTH	Normal intelligence
Primary failure of tooth eruption	125350	AR or AD	Loss of function or haploinsufficiency	n.a.	n.a.	n.a.	Primary failure of eruption	n.a.	n.a.	n.a.
Murk Jansen metaphyseal chondrodysplasia	156400	AD	Gain of function	n.a.	Severe postnatal short stature	Brachycephaly, hypertelorism, micrognathia.	Malposition	Generalized osteopenia, pathologic fractures, short bowed limbs, clinodactyly, short clubbed fingers	Hypercalcemia, hypophosphatemia, low or absent PTH	Waddling gait, deafness

Modified from OMIM clinical synopses.

iPPSD, inactivating PTH/PTHrP signalling disorder; AR, autosomal recessive; PTH, parathyroid hormone; n.a., not available; AD, autosomal dominant.

Herein, we report for the first time a child presenting PTH resistance, manifesting with seizures as first symptoms, due to a homozygous likely pathogenic variant in *PTH1R* gene. We delineate the extended phenotype comprising ossification, teeth and skull anomalies, and we add evidence that PTH resistance is part of a fifth iPPSD1 phenotype.

## Case Description

The proband is the third child of consanguineous (first cousins) parents originating from Turkey. He has two healthy siblings. There is no known history of endocrine disorder or epilepsy in the pedigree.

He was born by primary cesarean delivery because of breech presentation, at 39 weeks of gestation, after an uneventful pregnancy with a normal gestational diabetes screening test. He had an excellent neonatal adaptation. At birth, his weight was 4.440 kg (+2.2 SD, macrosomia), his height was 55 cm (+ 2.1 SD) and his head circumference was 36 cm (+1.0 SD). The mother was diagnosed with type 2 diabetes, treated with insulin injections, a few weeks after the delivery. Proband’s medical history comprised virus-induced airway hyperresponsiveness, gastroesophageal reflux and iron deficiency anemia, treated with inhaled salmeterol-fluticasone, omeprazole and iron supplementation, respectively. He exhibited no feeding/sleeping problem but mild neurodevelopmental delay (the sitting position without support was acquired at 10 months).

**Figure 1:**
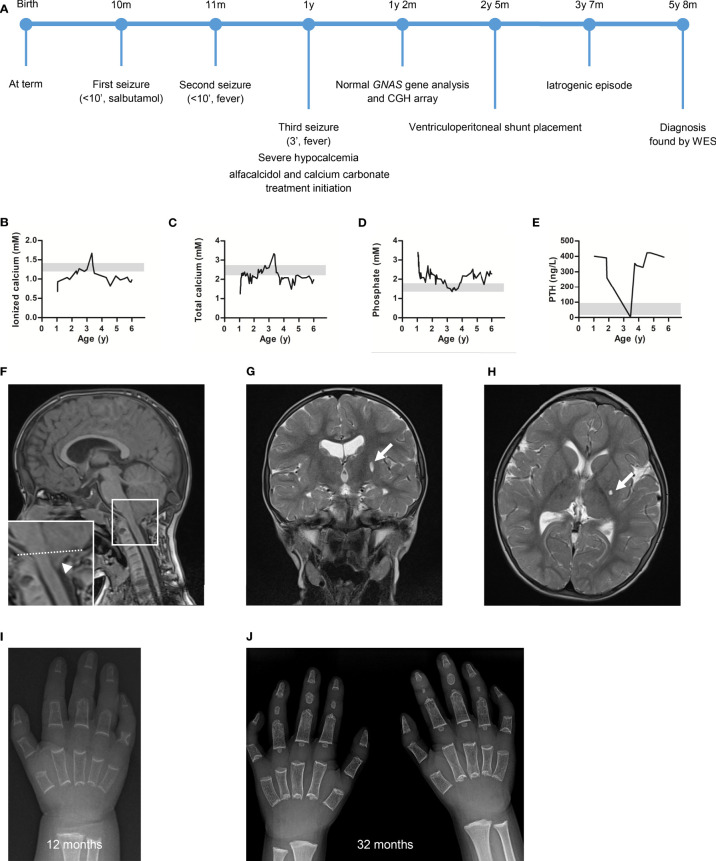
The patient presented with severe hypocalcemia and hyperphosphatemia which improved after treatment initiation. The work-up highlighted an Arnold-Chiari type 1 malformation (CM1) and delayed ossification on brain magnetic resonance imaging (MRI) and hands X-ray, respectively. **(A)** Overview of the patient’s diagnostic and therapeutic journey. Plasma **(B)** ionized calcium, **(C)** total calcium, **(D)** phosphate and **(E)** parathyroid hormone (PTH) concentrations measured at diagnosis and during follow-up. Of note, the dramatic PTH level decrease was associated with hypercalcemia resulting from an iatrogenic event (excessive alfacalcidol administration). Grey zone highlights normal value range. **(F)** Sagittal T1-weighted, **(G)** transversal T2-weighted, and **(H)** coronal T2-weighted brain MRI highlighting the CM1 (arrowhead, tonsillar herniation measured 10 mm below the drawn McRae line) and a 6 mm lacune in the posterior part of the left lentiform nucleus (arrow, stable on control MRI 6 months later). Hand X-ray at **(I)** 12 months and **(J)** 32 months of age showing diffuse osteopenia, sparse trabeculae, absence of carpal bones ossification, short metacarpal bones (only described at 32 months), absence (or hypotrophy) of the medial phalange ossification centers, cone-shaped distal phalanges and clinodactyly of the fifth digit.

At 10 months of age, during a short course of salbutamol treatment, he presented an acute episode with generalized (axial and peripheral) hypertonia, vomiting and loss of consciousness during less than 10 minutes. Two weeks later, he presented similar symptoms during a febrile episode associated with an upper respiratory tract infection. When he was 1 year old, he was referred to our center after a third shorter (3 minutes) episode with perioral cyanosis and drooling but no fever. All three episodes resolved spontaneously and the third one was followed by a postictal state (see timeline in **Figure 1A**). No access to toxic drug nor history of head trauma was reported.

Clinical examination of the patient showed enlarged anterior and posterior fontanelles associated with mild facial dysmorphic features including a round face, arched eyebrows, wide nasal bridge with telecanthus, bulbous nose and mild retrognathia. Bilateral clinodactyly of the fifth digit were noted without clear shortening of the 4^th^ and 5^th^ metacarpal bones. The rest of the clinical and neurological examination was unremarkable. The patient exhibited normally implanted nipples.

## Diagnostic Assessment

The biological work-up (**Figures 1B–E**) highlighted hypocalcemia (ionized calcium: 0.68 mM; nl 1.2-1.4 and total calcium <1.25 mM; nl 2.2-2.7), hyperphosphatemia (3.39 mM; nl 1.36-1.74), elevated PTH level (401 ng/L; nl 14-72), and normal levels of 25-OH-vitamin D (34.9 ng/L; nl 30-100) and magnesium (0.71 mM; nl 0.65-1.05). Thyroid function, cardiac ultrasound and electroencephalogram were normal. In the absence of chronic kidney disease, these results were compatible with a diagnosis of PTH resistance. The differential diagnosis of PTH resistance is given in **Table 2**. The patient was treated accordingly, with calcium carbonate titrated up to 800 mg 3x/d (5.4 mmol Ca^++^/kg/d) and alfacalcidol 1 μg 1x/d, leading to rapid bio-clinical improvement.

**Table 2:** PTH resistance differential diagnosis.

	25-OH-vitamin D levels	Kidney function	AHO	Erythrocyte Gsα activity	cAMP urinaryresponse to PTH	Multi-hormone resistance	Gene involved
iPPSD2	Normal	Normal	+	**↓**	**↓**	+	*GNAS* (genetic defect)
iPPSD3	Normal	Normal	±	Normal/↓	**↓**	±	*GNAS* (epigenetic defect)
iPPSD4	Normal	Normal	±	Normal	Normal	±	*PRKAR1A*
iPPSD5	Normal	Normal	±	Normal	Normal	±	*PDE4D*
Vitamin D deficiency	**↓**	Normal	–	n.a.	n.a.	n.a.	n.a.
Worsening of chronic kidney disease	Normal	**↓**	–	n.a.	n.a.	n.a.	n.a.

PTH, parathyroid hormone; AHO, Albright’s hereditary osteodystrophy; cAMP, cyclic adenosine monophosphate; iPPSD, inactivating PTH/PTHrP signalling disorder; +, present; ↓, low; ±, not alway present; -, absent; n.a., not applicable.

Brain magnetic resonance imaging (MRI) showed a 6 mm gap in the posterior part of the left lentiform nucleus and an Arnold-Chiari type 1 malformation (CM1) (**Figures 1F–H**). Control MRI performed 6 months later showed no progression of the gap and highlighted “benign” intracranial hypertension signs and bilateral transverse sinus stenosis with collateral vessels development. Dilated fundus examination confirmed a high probability of intracranial hypertension and the patient underwent ventriculoperitoneal shunt placement. Hand x-rays showed diffuse delayed bone age, osteopenia, sparse trabeculae, absence of carpal bones ossification, short metacarpal bones, absence (or hypotrophy) of the medial phalange ossification centers, cone-shaped distal phalanges and clinodactyly of the fifth digit (**Figures 1I, J** **)**. No soft tissue ossification was noted on skeleton x-ray.

**Figure 2:**
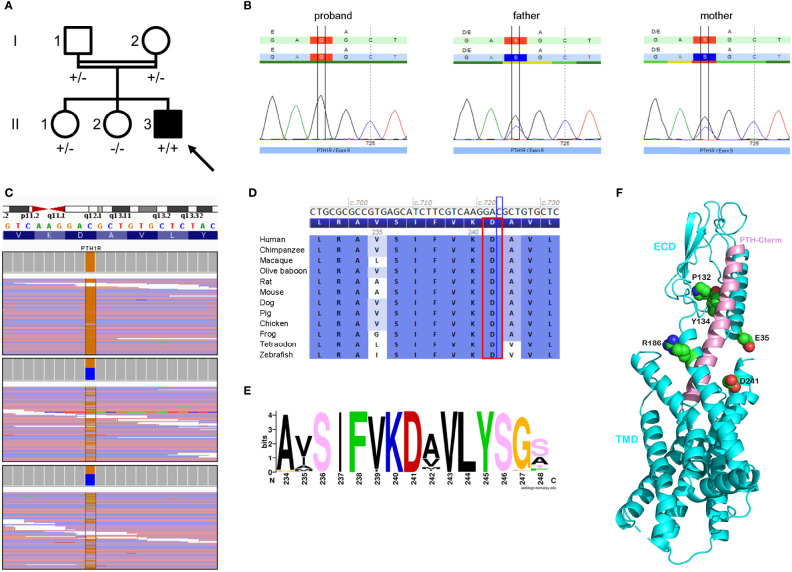
Genealogy, sequencing and bioinformatics supported the pathogenicity of the c.723C>G p.(Asp241Glu) variant in the *PTH1R* gene (NM_000316.3). **(A)** Pedigree of the family showing the index case (arrow), who is homozygous for the *PTH1R* variant (+/+), his unaffected (white symbol) parents (I.1 and I.2) and sister (II.1), who are heterozygous carriers of the variant (+/-), and his unaffected sister (II.2) who does not carry the *PTH1R* variant (-/-). **(B)** Sanger chromatograms confirming the homozygous and heterozygous status of our patient and his parents. **(C)** Next-generation sequencing uncovering the homozygous transversion, cytosine to guanine, with a sequencing depth of 132 x in the proband (upper panel) compared to heterozygous carrier status in his parents (lower panels). **(D)** Protein sequence alignment of 12 vertebrates species highlighting conservation of the amino-acid residue (red rectangle) up to the zebrafish. **(E)** WebLogo analysis of the region 234-248 among PTH1R sequences showing the high conservation of the position D241. The picture was produced *via* the web server weblogo with 979 protein sequences collected by a Blast search in the UniRef100 database and aligned with the clustal2.1 algorithm. **(F)** Ribbon representation of the crystal complex structure (PDB 6FJ3) between the PTH1R protein (in blue) and its peptide ligand (parathyroid hormone, in pink) highlighting the patient’s affected residue (D241, labeled and depicted in sphere representation) in close vicinity with the peptide hormone. Four other affected PTH1R residues previously reported in iPPSD1 patients are also labeled and showed in the 3D structure (see **Supplementary Table 3**). In residue sphere representation, atoms of carbon, oxygen and nitrogen are colored in green, red and blue, respectively. The C-terminal extremity of peptide hormone PTH is indicated. TMD and ECD mean transmembrane domain and extra-cellular domain.

Comparative genomic hybridization array (180K) and epilepsy targeted gene panel detected no pathological copy number variation or pathogenic variant, respectively. Sanger sequencing and methylation-specific multiplex ligation-dependent probe amplification (MS-MLPA) of the *GNAS* locus revealed no pathogenic variant, large deletion or abnormal methylation pattern. Trio-based whole exome sequencing (WES) revealed a homozygous substitution c.723C>G p.(Asp241Glu) in exon 9 of the *PTH1R* gene (NM_000316.3) which was confirmed by Sanger sequencing (ClinVar SCV001984749). Both parents carried the substitution in heterozygous state (**Figures 2A–C**). The variant was not found in large databases (gnomAD, ESP, ClinVar) or in our in-house exome database. The variant affects an amino-acid residue located in the N-terminal part of the second transmembrane helix of PTH1R class B G-protein-coupled receptor (GPCR), and found in interaction with the peptide hormone in the complex crystal structure of PTH1R (8) (**Supplementary Table 3**). The residue D241 is highly-conserved, both between species and among *H. sapiens* paralogs (**Figures 2D–F**). The vast majority of bioinformatics tools (10/12) recognize the variant as deleterious. According to the American College of Medical Genetics criteria, this variant was considered as likely pathogenic (PM2, PM3, PP1, PP2, PP3 and PP4) (9).

Under treatment, calcemia and phosphoremia improved but did not normalize, and PTH levels remained elevated (**Figures 1B–E**). At 38 months, renal ultrasound showed grade 2 medullary nephrocalcinosis in the context of hypercalcemia, hypophosphoremia, abolished PTH secretion, and chronic asymptomatic hypercalciuria resulting from an iatrogenic event (overtreatment). The treatment was adjusted (calcium carbonate 200 mg 1x/d, alfacalcidol 0.25 μg 1x/d) to achieve a near normal calcemia (2-2.4 mM) and avoid hypercalciuria. Nephrocalcinosis partially resolved on ultrasound performed 2 years later.

When he was 5 year-old, he was treated for multiple agenesis of primary teeth and delayed teeth eruption.

At last follow-up, at the age of 5 years and 8 months, his weight, height, body mass index, and head circumference were 23.5 kg (P75), 112.5 cm (P25), 18.6 kg/m² (P90-P97, overweight), and 51.5 cm (P60), respectively. He followed normal education and he was well integrated. He was on low-phosphorus diet because of persisting hyperphosphatemia, and he received calcium carbonate 400 mg 1x/d and calcitriol 0.25 µg 1x/d. There was no recurrence of seizure since the biological diagnosis was made.

## Discussion

Bi-allelic *PTH1R* pathogenic variants have been associated with: 1. Blomstrand chondrodysplasia (BCD, MIM 215045), a lethal chondrodysplasia presenting with hydrops fetalis, increased bone density and, in some cases, absence of nipples (10–13), 2. Eiken Syndrome (ES, MIM 600002), in patients with delayed ossification (14, 15), normal calcium and PTH levels and short stature, and 3. primary failure of tooth eruption (16) (PFTE, MIM 125350, a feature also present in ES (17, 18)). Interestingly, in addition to PTH resistance, our patient exhibited symptoms in common with ES including delayed ossification, PFTE, febrile seizures (17) and CM1 (18). *PTH1R* pathogenic variants are dispersed all along the gene and the protein domains. Until now, no genotype-phenotype correlation could be drawn (17).

As it was shown in a mouse model (19), we postulate that the various phenotypes might reflect a different impact of the variants on adenyl cyclase (calcium homeostasis) and phospholipase C (embryonic endochondral bone development), two intracellular effectors activated by PTH1R in response to PTH. Here we report on a 10-month-old boy, born from consanguineous parents, presenting with seizures in the context of hypocalcemia secondary to PTH resistance. Trio-based WES found a homozygous missense variant c.723C>G p.(Asp241Glu) in *PTH1R* gene (NM_000316.3) leading to the diagnosis of iPPSD1.

Zhao et al. showed that the D241 residue was highly conserved among class B GPCRs and that it was involved in the peptide binding pocket of PTH1R (20). Considering the 3D localization of D241 residue and the “two domain binding model” for class B GPCRs (21), we postulate that the p.(Asp241Glu) substitution alters the second step of the binding process in which N-terminal part of the peptide hormone binds to the juxtamembrane region of the PTH1R, mediating thereby the receptor activation. Of note, although the p.(Asp241Glu) substitution does not modify the physicochemical properties, the resulting small expansion in side-chain length may presumably affect the precise binding of ligand to PTH1R. Hence, the homozygous variant may lead to alteration of PTH signaling causing calcium homeostasis impairment and ossification disturbances.

Our patient exhibited the classical biological alterations seen in PTH resistance (i.e. hypocalcemia, hyperphosphatemia and elevated PTH levels). *PTH1R* gene mutations have been shown to reduce the cAMP response to PTH *in vitro* (22). In PHP patients, cAMP response to exogenous PTH was formerly used to distinguish between different types of PHP. To our knowledge, this has never been evaluated in patients carrying *PTH1R* pathogenic variants. In our case, the diagnosis of iPPSD1 was made through WES and it was decided not to perform an invasive test (i.e. PTH stimulation test) to ascertain the diagnosis.

Guerreiro et al. reported an adult patient, born from consanguineous parents, presenting drug-resistant epilepsy and a biological work-up compatible with PTH resistance. WES discovered homozygous *PTH1R* pathogenic variant, both parents being heterozygous carrier. Two siblings, one being asymptomatic and the other with speech problem at age 50, were also homozygous for the *PTH1R* variant. Basal ganglia calcification and PTH elevation was shown in all three siblings (7). These 3 patients might reflect an adult, milder, presentation of PTH resistance caused by bi-allelic *PTH1R* pathogenic variant.

CM1 was detected after the genetic diagnosis was made in our patient. Obviously, since parental consanguinity increases homozygosity regions in the progeny (23), we cannot exclude that some clinical features presented by our patient may be due to additional pathogenic variants. We performed a second analysis of the WES results to filter the variants with the Human Phenotype Ontology HP:0002308, corresponding to Arnold-Chiari malformation. We found no additional pathogenic or likely pathogenic variants. Interestingly, CM1 was previously reported in a patient with Eiken syndrome (ES) (18) and in 3 patients with PHP1A (24–26). The underlying mechanisms have not been elucidated yet but growth hormone pathway alteration might be involved in CM1 associated with PHP1A (25, 27).

In conclusion, we report a homozygous likely pathogenic variant in *PTH1R* gene and we provide a deep phenotypic and bioinformatics characterization. Based on this report and the literature (7, 10–12, 14–18, 22, 28), it emerges that the iPPSD1 phenotypes, associated with bi-allelic *PTH1R* pathogenic variants, form a large spectrum from chondrodysplasia to PTH resistance. Along with endocrinological anomalies, the patient presented with ossification anomalies, Arnold-Chiari type 1 malformation and primary failure of tooth eruption. His phenotype locates between ES, PTFE and the PHP1B patient reported by Guerreiro et al. (7), and it could reflect a fifth iPPSD1 phenotype. Taken together, data argue the iPPSD1 as a large spectrum of overlapping phenotypes.

## Patient Perspective

The genetic diagnosis confirmed the appropriateness of the patient’s treatment, refined his follow-up and allowed the family to receive precise genetic counseling. The patient confessed that the low-phosphorus diet is the most difficult part of the treatment.

## Data Availability Statement

The original contributions presented in the study are included in the article/**Supplementary Material**. Further inquiries can be directed to the corresponding author.

## Ethics Statement

Ethical review and approval was not required for the study on human participants in accordance with the local legislation and institutional requirements. Written informed consent to participate in this study was provided by the participants’ legal guardian/next of kin.

## Author Contributions

TD: conceptualization, methodology, data curation, original draft writing, review and editing; RW: data curation, formal analysis, methodology, original draft writing, review and editing; GS: data curation, formal analysis, review and editing; JD: data curation, formal analysis, review and editing; FB: data curation, formal analysis, review and editing; CI: data curation, formal analysis, review and editing; OM: data curation, formal analysis, review and editing; DK: data curation, formal analysis, review and editing; VB: data curation, formal analysis, methodology, supervision, review and editing; IM: conceptualization, methodology, original draft writing, supervision, review and editing; All authors agree to be accountable for the content of the work. All authors contributed to the article and approved the submitted version.

## Funding

RW is a Research Associate with the Belgian National Funds for Scientific Research (FRS-FNRS). Funding covering publication fees received from the Conseil Scientifique de l’Institut de Pathologie et Génétique.

## Conflict of Interest

The authors declare that the research was conducted in the absence of any commercial or financial relationships that could be construed as a potential conflict of interest.

## Publisher’s Note

All claims expressed in this article are solely those of the authors and do not necessarily represent those of their affiliated organizations, or those of the publisher, the editors and the reviewers. Any product that may be evaluated in this article, or claim that may be made by its manufacturer, is not guaranteed or endorsed by the publisher.
